# Sudden re-emergence of *Streptococcus pyogenes* subtype *emm*3.93 in Spain, 2023-2024

**DOI:** 10.1016/j.nmni.2026.101794

**Published:** 2026-06-13

**Authors:** Villalón Pilar, Medina-Pascual María José, Garrido Noelia, Valiente Mónica, Monzón Sara, Varona Sarai, Valdezate Sylvia

**Affiliations:** a Laboratorio de Referencia e Investigación en Taxonomía, Centro Nacional de Microbiología, Instituto de Salud Carlos III, Majadahonda, 28220, Spain; b Unidad de Bioinformática, Unidades Centrales Científico-Técnicas, Instituto de Salud Carlos III, Majadahonda, 28220, Spain

**Keywords:** *Streptococcus pyogenes*, Type *emm*3, Subtype *emm*3.93, Outbreak, Chromosomal arrangement, Prophage, Exotoxin

## Abstract

**Background:**

The re-emergence of *Streptococcus pyogenes* genotype *emm*3 in Spain after the COVID-19 pandemic was driven by the expansion of the rare subtype *emm*3.93 which caused an outbreak between November 2023 and June 2024. This study aimed to characterize the microbiological features of emm3 isolates recovered during 2020–2024, incorporating clinical and epidemiological data.

**Methods:**

A total of 138 *emm*3 strains were studied. *emm* and exotoxin genes were typed by PCR-sequencing. Antimicrobial susceptibility was undertaken by E-test. Ten *emm*3.93 genomes were further investigated by whole genome sequencing, and chromosomal arrangements were determined by long acute LA-PCR.

**Results:**

Type *emm*3 was associated with sepsis, and children and elderly people were the most vulnerable. Subtype *emm*3.93 was detected in 114/138 *emm*3 strains. The *emm*3 population was susceptible to penicillin G, tetracycline, erythromycin and clindamycin. All genomes belonged to the sequence type ST315 and carried the six characteristic prophages Φ315.1, Φ315.2, Φ315.3, Φ315.4, Φ315.5 and Φ315.6; and 4/10 genomes also harboured ΦspeC-spd1. The predominant chromosomal arrangement (8/10 genomes) displayed an inversion around the terminus of replication, and 2/10 genomes were like the *emm*3 reference genome MGAS315. Phylogenetic study grouped 7/10 genomes into the “Spanish clade”; and 3/10 genomes clustered with international strains, which evidenced global dissemination of the emergent *emm*3.93 subtype.

**Conclusions:**

The 2023-2024 outbreak was caused by the emergent clone *emm*3.93/*spe*A-*spe*G-*spe*K-*sme*Z-*ssa*/ST315, representative of contemporary hypervirulent *emm*3 lineages.

## Highlights

• GAS type *emm*3 re-emerged in 2023 after a silent period following COVID-19 pandemic.
• GAS *emm*3.93 has become established as the predominant *emm*3 subtype in Spain.
• Rapid spread of *emm*3.93 caused an outbreak of international scope in 2023-2024.
• Subtype *emm*3.93 is representative of contemporary hypervirulent GAS *emm*3 clones.
• Surveillance is essential to prevent and control the invasive GAS disease.

## 1 Introduction

*Streptococcus pyogenes*, or group A *Streptococcus* (GAS), is classified into *emm* types based on the hypervariable 5′ region of the *emm* gene encoding the surface M protein, an important factor for bacterial survival against host defenses. *emm* typing, performed by molecular methods or whole-genome sequencing, is essential for GAS strains characterization [1,2]. Invasive *Streptococcus pyogenes* infection (iGAS) includes a wide spectrum of diseases with high morbidity and mortality, such as bacteremia/sepsis, pneumonia, arthritis, cellulitis, necrotizing fasciitis (NF), and streptococcal toxic shock syndrome (STSS) [3]. A limited number of *emm* types, formerly referred to as serotypes, account for most iGAS cases in developed countries [[4], [5], [6]].

In the last five years, iGAS epidemiology has undergone relevant changes. During the COVID-19 pandemic, social distancing and respiratory measures were implemented to limit viral transmission, resulting in a marked decline in iGAS incidence for two and a half years. In November 2022, a sudden increase in iGAS cases was reported in multiple countries [7,8]. This marked the beginning of an international iGAS outbreak, mainly associated with *emm*1 and *emm*12 GAS strains [[9], [10], [11]], which persisted until the first trimester of 2023.

*emm*3 has been frequently associated with iGAS, contributing to multiple outbreaks, and the genomic features underlying its hypervirulence have been deeply studied in some strains [12,13]. However, *emm*3 was absent during the 2022–2023 global outbreak [[9], [10], [11]]. In November 2023, the Spanish Surveillance Program for Invasive GAS Infection (SPIGAS) [14] detected a sudden increase in *emm*3 cases driven by the expansion of the rare subtype *emm*3.93.

This research primarily aims to study the microbiological features of the re-emergent GAS type *emm*3 during the 2020-2024 year-period in Spain, with clinical and epidemiological data included as complementary context.

## 2 Materials and methods

### 2.1 Study design

A retrospective observational study was conducted using *emm*3 GAS strains analyzed by SPIGAS during 2020–2024. SPIGAS is a passive, nationwide laboratory-based program. GAS strains are voluntarily submitted by Spanish hospitals to the *Centro Nacional de Microbiología–Instituto de Salud Carlos III* (CNM-ISCIII) for characterization [14]. The microbiological criterion for invasive disease was isolation of GAS from normally sterile body sites. Reported epidemiological and clinical data included age, sex, geographical origin, date of isolation, sample type, and clinical syndrome. Only one isolate per clinical case was included.

### 2.2 Basic microbiological typing of *emm*3 strains

*emm* gene typing was undertaken by sequencing the 180 nucleotides of the hypervariable 5′-end using the *Centers for Disease Control and Prevention* protocols [2]. The presence of the exotoxin genes *spe*A, *spe*C, *spe*G, *spe*H, *spe*J, *sme*Z and *ssa* [15], and the macrolides-resistance genes *mef*A [16], *erm*B [17], *erm*TR [18] and *erm*T [17] was determined by PCR. Susceptibility to penicillin G, tetracycline, erythromycin and clindamycin was undertaken by E-test and interpreted according to *The European Committee on Antimicrobial Susceptibility Testing* (EUCAST) criteria [19]. Phenotypes of resistance to macrolides, lincosamides and streptogramin B were determined by the erythromycin-clindamycin double-disk test [20].

### 2.3 Statistical study

Statistical analysis focused on the 2023-2024 period. Associations between categorical variables were examined using Fisher's exact test. Significance was set at *p* ≤ 0.05. All calculations were made using Stata v.17 software.

### 2.4 Whole genome sequencing (WGS) analysis

WGS of ten *emm*3.93 strains was performed (Table 1). Paired-end libraries were made using the Nextera-XT DNA Library Preparation Kit (Illumina 1.9) and sequencing was performed using the Illumina NovaSeq platform. Removing low-quality regions was done with Fastp v.0.23.4 [21]. The quality metrics were obtained applying FastQC v.0.12.1 software. Sample purity was assessed using Kmerfinder v.3.0.2 [22]. *De novo* assembly of short reads was resolved with Unicycler v.0.4.8 [23], assembly quality was performed with QUAST v.5.2.0 [24], and genomes were annotated with Prokka v.1.14.6 [25].

**Table 1:** Genomic and clinical-epidemiological features of three reference *Streptococcus pyogenes* type *emm*3 strains and of ten strains representative of the resurgence of subtype *emm*3.93 in Spain, 2023-2024.

Strain	MGAS315	SSI-1	MGAS10870	20231359	20240005	20240018	20240054	20240154	20240173	20240174	20240285	20240356	20240409
Genomic features													

Acc. no. a	NC_004070	NC_004606	CP067090	SAMN53128930	SAMN53128931	SAMN53128932	SAMN53128933	SAMN53128934	SAMN53128935	SAMN53128936	SAMN53128937	SAMN53128938	SAMN53128939
Length (bp)	1,900,521	1,894,275	1,863,912	1,859,289	1,842,134	1,869,786	1,824,241	1,829,260	1,850,802	1,837,893	1,838,236	1,855,015	1,839,612
G + C (%)	38.5	38.5	38.5	38.35	38.39	38.36	38.37	38.38	38.34	38.38	38.38	38.35	38.39
Inversion *ori*	No	Yes	No	No	No	No	No	No	No	No	No	No	No
Inversion *ter*	No	Yes	No	Yes	Yes	Yes	Yes	Yes	Yes	No	No	Yes	Yes
Phages no.	6	6	5	7	6	7	6	6	7	6	6	7	6
Φ315.1	Yes	Yes	No	Yes	Yes	Yes	Yes	Yes	Yes	Yes	Yes	Yes	Yes
Φ315.2	Yes	Yes	No	Yes	Yes	Yes	Yes	Yes	Yes	Yes	Yes	Yes	Yes
Φ315.3	Yes	Yes	Yes	Yes	Yes	Yes	Yes	Yes	Yes	Yes	Yes	Yes	Yes
Φ315.4	Yes	Yes	Yes	Yes	Yes	Yes	Yes	Yes	Yes	Yes	Yes	Yes	Yes
Φ315.5	Yes	Yes	Yes	Yes	Yes	Yes	Yes	Yes	Yes	Yes	Yes	Yes	Yes
Φ315.6	Yes	Yes	Yes	Yes	Yes	Yes	Yes	Yes	Yes	Yes	Yes	Yes	Yes
ΦspeC-spd1	No	No	Yes	Yes	No	Yes	No	No	Yes	No	No	Yes	No

Clinical and epidemiological features													

Age (y)	-	-	-	77	45	74	50	39	38	9	3	12	3
Sex	-	-	-	M	F	F	M	F	M	M	F	F	M
Sample	-	-	-	Blood	Blood	Blood	Blood	Blood	Blood	Blood	Blood	Blood	Blood
Syndrome	-	-	-	Pne, Sep	Ost, Sep	STSS	Sep	Sep	Pne, Sep	SF, Sep	Ost, Sep	Sep	Sep

Abbreviations: M, male; F, female; Ost, osteomyelitis; Pne, pneumonia; Sep, sepsis; SF, scarlet fever; STSS, streptococcal toxic shock syndrome.

a DDBJ/ENA/GenBank bioproject PRJNA1358835.

### 2.5 Chromosomal arrangements and prophage content of subtype *emm*3.93

Two large chromosomal inversions around the origin (*ori*) and the terminus (*ter*) of replication were determined by long acute LA-PCR (Takara) using primers described by Beres et al. [26]. The *emm*3 strains MGAS315 (GenBank accession. no. NC_004070) [12], and SSI-1 (NC_004606) [13] were used as references (Table 1).

Prophages were detected with PHASTEST [27]. The presence of the prophage carrying *spe*C and *spd*1 (ΦspeC-spd1 hereinafter) was confirmed using Proksee [28] through alignment against the reference genome MGAS10870 (CP067090) (Table 1).

### 2.6 Searching for virulence and resistance genes

The presence of virulence and resistance genes was analyzed with Ariba v.2.14.7 [29] by using the Virulence Factor Database (VFDB, https://www.mgc.ac.cn/VFs/) and the CARD database (https://card.mcmaster.ca/), respectively. Multilocus sequence typing (MLST) was carried out according to the *S. pyogenes* public database PubMLST (https://pubmlst.org/bigsdb?db=pubmlst_spyogenes_seqdef) [30]. Plasmids were searched with PlasmidFinder 2.1 tool (https://cge.food.dtu.dk/services/PlasmidFinder/).

### 2.7 Phylogenetic study of *Streptococcus pyogenes* type *emm*3

Alignment of 9769 nucleotide polymorphic sites (SNPs) from the core genome against the reference MGAS10870 was done using Snippy v.4.6.0 (https://github.com/tseemann/snippy). Recombination sites were filtered with Gubbins v.3.3.5 [31]. Relationships between strains were estimated in a maximum-likelihood phylogenetic tree using the IQ-TREE v.2.1.4 algorithm [32]. The analysis included 178 *emm*3 strains: ten *emm*3.93 strains from this study, 104 *emm*3.93 strains reported by Davies et al. [33], and 64 *emm*3 (non-*emm*3.93) strains available at the *National Center for Biotechnology Information* (NCBI) (https://www.ncbi.nlm.nih.gov/datasets/genome/?taxon=301448, last accessed 3 March 2026). The tree was rooted with the *emm*1 reference genome MGAS5005 (NC_007297) and visualized with MegaX Tree Explorer software v.12.2.4.

### 2.8 Ethical statement

No identifiable human data were used. This study focused on bacterial strains submitted to a public national reference laboratory (CNM-ISCIII) for microbiological typing. Ethical approval was not required.

## 3 Results

### 3.1 Epidemiological and clinical data of *emm*3 infection

**Fig. 1:**
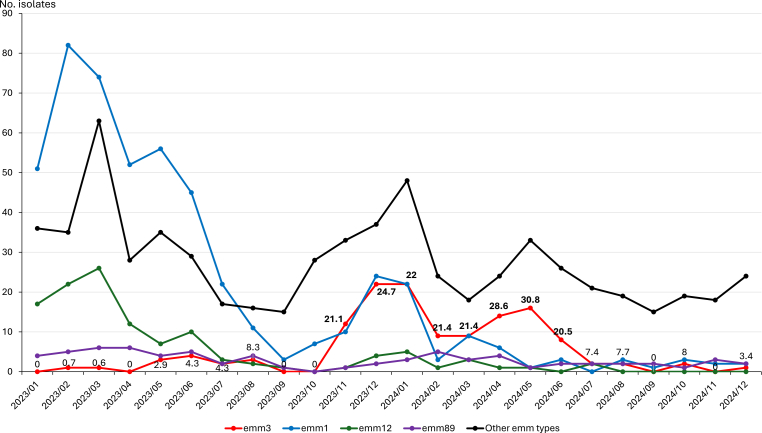
Outbreak of *Streptococcus pyogenes* type *emm*3 from November 2023 to June 2024 in Spain. The line graph represents time series of *emm*3, *emm*1, *emm*12, *emm*89 and the remaining 73 *emm* types during the 2023-2024 year-period. The percentage of *emm*3 isolations is indicated over the entire period and the outbreak months are highlighted in bold.

*emm*3 was detected in 138/1815 GAS strains typed during 2020-2024. Three *emm*3 strains were collected in 2020-2022, 50 in 2023 and 85 in 2024. Fig. 1 represents the *emm*3 outbreak during 2023-2024.

Cases of *emm*3 infection were reported from 14 of 17 Spanish Autonomous Communities. No differences were observed between men (n = 73, 52.9%) and women (n = 65, 47.1%). The age range, median, and mode were 0–92, 38, and 3 years, respectively. The highest prevalence was observed in children aged 0–9 years (31.9%) and adults aged ≥70 years (18.8%).

*emm*3 strains were isolated from blood (n = 72, 52.2%), abscess puncture (18, 13.0%), otic exudate (17, 12.3%), pleural fluid (8, 5.8%), and other clinical samples (23, 16.7%). iGAS affected 116/138 (84.1%) patients, while 22/138 (15.9%) corresponded to superficial infections like otitis, pharyngitis and vaginitis. Multiple clinical syndromes were reported in 32 patients. The syndromes involved were sepsis (64 cases), pneumonia (25), otitis (19), abscesses (16), osteoarticular infections (OAI) (10), STSS (7), pharyngitis (7), cellulitis (6), puerperal fever (5), NF (3), meningitis (3), mastoiditis (2), scarlet fever (2), and other infections (4). Only sepsis was significantly associated with *emm*3 infection (*p* ≤ 0.05). Table 2 summarizes the statistics of clinical syndromes. Supplementary Table 1 shows the temporal distribution of clinical syndromes caused by *emm*3 during 2020-2022 and 2023-2024.

**Table 2:** Clinical syndromes caused by *Streptococcus pyogenes* type *emm*3 in Spain, 2023-2024.

Clinical syndrome^a^	No. *emm*3 cases (%)	No. other GAS cases (%)^b^	p value	95% CI	OR
Sepsis	62 (15.2)	346 (84.8)	0.002	0.018 - 0.096	1.7
Pneumonia	25 (12.4)	176 (87.6)	0.469	−0.031 – 0.065	1.2
Otitis	19 (16.8)	94 (83.2)	0.059	−0.008 – 0.134	1.7
Abscess	16 (10.1)	142 (89.9)	0.789	−0.060 – 0.040	0.9
OAI	10 (16.7)	50 (83.3)	0.144	−0.037 -0.155	1.7
Pharyngitis	7 (7.8)	83 (92.2)	0.387	−0.092 – 0.023	0.7
STSS	6 (8.7)	63 (91.3)	0.694	−0.092 – 0.044	0.8
Cellulitis	6 (4.4)	130 (95.6)	0.009	−0.111 to −0.034	0.4
Puerperal fever	5 (26.3)	14 (73.7)	0.045	−0.042 – 0.355	3.0
NF	3 (6.3)	45 (93.8)	0.356	−0.119 – 0.021	0.5
Meningitis	3 (18.8)	13 (81.3)	0.407	−0.114 – 0.270	1.9
Mastoiditis	2 (8.3)	22 (91.7)	1	−0.139 – 0.085	0.7
Scarlet fever	2 (10.0)	18 (90.0)	1	−0.143 – 0.122	0.9
Peritonitis	1 (14.3)	6 (85.7)	0.558	−0.227 – 0.293	1.4
SWI	1 (2.2)	45 (97.8	0.053	−0.136 to −0.046	0.2
Vaginitis	1 (3.1)	31 (96.9)	0.247	−0.143 to −0.018	0.3
Other^c^	0 (0.0)	259 (100)	-	-	-
Total	169 (11.0)	1368 (89.0)	-	-	-

Abbreviations: CI, confidence interval; OR, odds ratio; OAI, osteoarticular infection; STSS, streptococcal toxic shock syndrome; NF, necrotizing fasciitis; SWI, surgical wound infection.

a Multiple clinical syndromes in the same patient were analyzed as independent variables.

b *S. pyogenes* clinical syndromes caused by all *emm* types, except *emm*3.

c Other clinical syndromes not caused by *emm*3 strains.

### 3.2 Molecular typing and antimicrobial susceptibility testing

**Fig. 2:**
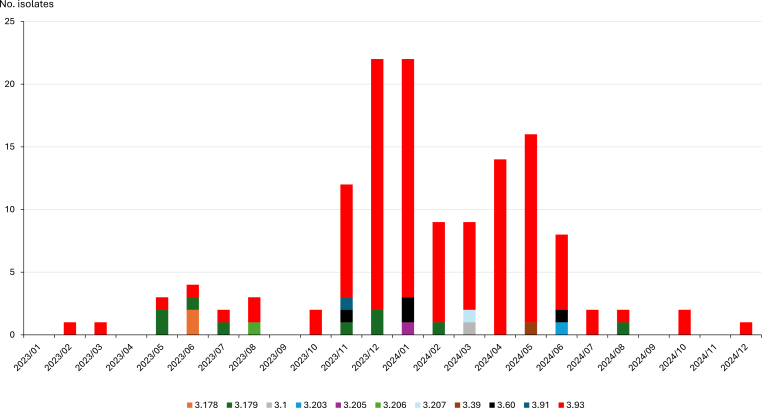
Temporal distribution of *emm*3 subtypes in the 2023-2024 year-period in Spain. Stacked bar chart that represents the number of strains of the eleven *emm*3 subtypes detected. The peak of *emm*3.93 was registered from November 2023 to June 2024.

**Fig. 3:**
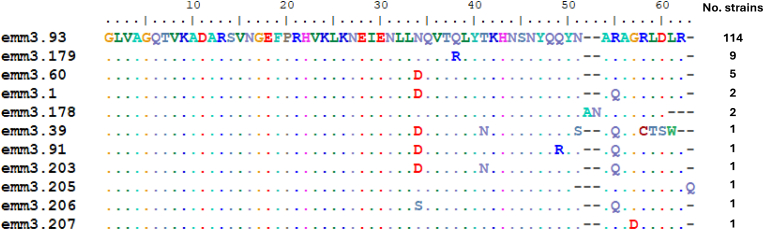
Amino acid sequences of the eleven *emm*3 subtypes detected in Spain in 2020-2024. The sixty amino acids of the hypervariable N-t end of protein M are represented. Alignment of sequences was done with BioEdit Editor [34]. Dots represent identical residues. The number of strains of each *emm*3 subtype is indicated.

Eleven *emm*3 subtypes were detected: *emm*3.93 (114 strains, 82.6%); *emm*3.179 (9, 6.5%); *emm*3.60 (5, 3.6%); *emm*3.1 and *emm*3.178 (2, 1.5% each); and *emm*3.39, *emm*3.91, *emm*3.203, *emm*3.205, *emm*3.206 and *emm*3.207 (1, 0.7% each). In November 2023, *emm*3.93 emerged and caused an eight-month outbreak. Fig. 2 represents the temporal distribution of *emm*3 subtypes in 2023-2024. Subtype *emm*3.1 -the most common in Spain before the COVID-19 pandemic- and subtype *emm*3.93 -the currently predominant subtype-differed by two non-synonymous nucleotide substitutions: G100A→Asp34Asn and A158G→Gln53Arg. Fig. 3 displays the protein sequences of the 11 *emm*3 subtypes.

**Fig. 4:**
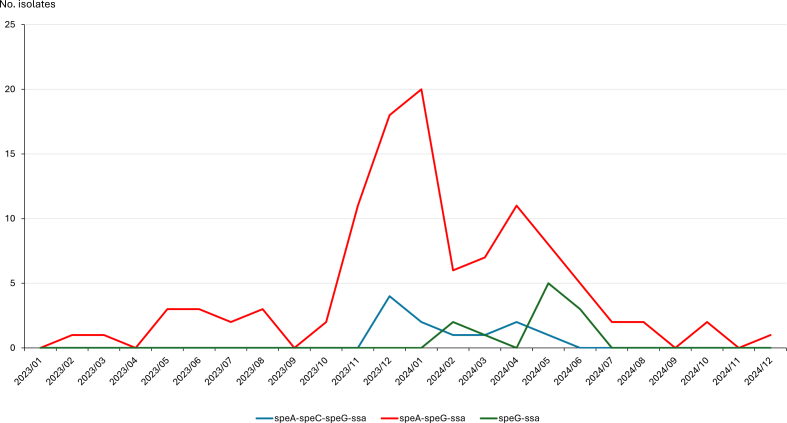
Temporal distribution of the most frequent *emm*3 exotoxin profiles detected by PCR in the 2023-2024 period in Spain. The majority profile *spe*A-*spe*G-*ssa*, and the minority *spe*A-*spe*C-*spe*G-*ssa* and *spe*G-ssa were involved in the outbreak.

The most prevalent exotoxin profile, *spe*A-*spe*G-*ssa*, was present in 111/138 strains. The minority profiles *spe*A-*spe*C-*spe*G-*ssa* and *spe*G-*ssa* were each detected in 11 strains. No statistically significant association was observed between exotoxin profiles and the clinical syndromes caused by *emm3*. Fig. 4 shows the temporal distribution of the most frequent exotoxin profiles during 2023-2024.

All strains were susceptible to penicillin G, tetracycline, erythromycin and clindamycin; except one *emm*3.93 strain resistant to erythromycin and clindamycin (MIC>256 mg/L). Table 3 shows the results of antimicrobial susceptibility for *emm*3 population.

**Table 3:** Antimicrobial resistance of 138 *Streptococcus pyogenes* type *emm*3 strains, Spain 2020-2024.

Antimicrobial agent	n^a^	%^a^	MIC_50_^b^	MIC_90_^b^	AMR gene (n)	Phenotype^c^ (n)
Penicillin G	0	0	0.008	0.012	ND	NA
Tetracycline	0	0	0.25	0.38	ND	NA
Erythromycin	1	0.7	0.094	0.19	*erm*B (1) d	cMLS_B_ (1) d
Clindamycin	1	0.7	0.094	0.19	*erm*B (1) d	cMLS_B_ (1) d

Abbreviations: MIC, minimum inhibitory concentration; AMR, antimicrobial resistance; ND, not detected; NA, not applicable; cMLS_B_, constitutive macrolide, lincosamide and streptogramin B phenotype; S, susceptible; R, resistant.

a Number (n) and percentage (%) of resistant strains.

b MICs are expressed in mg/L and interpreted according to EUCAST criteria [20]. Penicillin G: ≤0.25 S, >0.25 R. Tetracycline: ≤1 S, >1 R. Erythromycin: ≤0.25 S, >0.5 R. Clindamycin: ≤0.5 S, >0.5 R.

c Phenotype with respect to resistance to macrolides, lincosamides and streptogramin B.

d Resistance to erythromycin and clindamycin is concentrated in one co-resistant strain only.

### 3.3 Chromosomal arrangements and prophage content of subtype *emm*3.93

The chromosomal inversion around the *ori* of replication was not detected in any strain, whereas the inversion around the *ter* of replication was identified in 8/10 strains.

All ten strains carried the prophages Φ315.1, Φ315.2, Φ315.3, Φ315.4, Φ315.5 and Φ315.6; and four strains harboured the additional prophage ΦspeC-spd1. Table 1 summarizes the genomic features of three *emm*3 reference genomes and ten *emm*3.93 strains.

### 3.4 Virulence genomic features of subtype *emm*3.93

Six strains exhibited the *spe*A3-*spe*G-*spe*K-*sme*Z-*ssa* exotoxin-gene profile, whereas four strains showed the *spe*A3-*spe*C-*spe*G-*spe*K-*sme*Z-*ssa* profile.

All strains carried prophage-encoded genes for the streptodornases *spd*4 and *sdn*, and for the phospholipase A2 *sla*A. The DNAase gene *spd*1 was detected in strains harbouring the phage ΦspeC-spd1. No allelic variation was observed in any virulence gene.

All strains showed an identical *nag-slo* operon. Compared with the *emm*1 ancestral strain SF370 (NC_002737); the promoter harboured three polymorphisms −27A, −22G and −18T [35], and the *nag* gene (NAD glycohydrolase) showed a non-synonymous SNP A989G → Asp330Gly [36]. No polymorphisms were detected in the *slo* gene (streptolysin O).

MLST assigned the sequence type (ST) ST315 for all strains. Antimicrobial resistance genes and plasmids were not found.

### 3.5 *Streptococcus pyogenes* type *emm*3 phylogenetic relationships

**Fig. 5:**
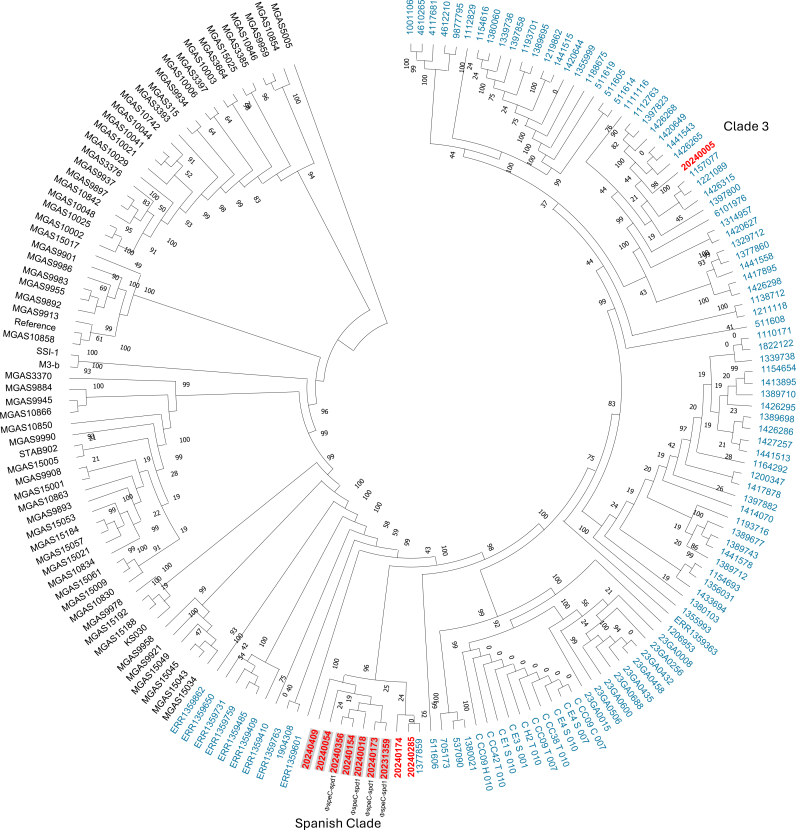
Maximum-likelihood phylogenetic tree obtained from the analysis of 9769 SNPs of the core genome of 178 *emm*3 strains. The alignment was done against the *emm*3 reference genome MGAS10870. The tree was rooted with the *emm*1 genome MGAS5005. Bootstrap values are indicated in branches. The ten *emm*3.93 genomes of this study are in bold red, and the “Spanish Clade” is highlighted in grey. One hundred and four *emm*3.93 genomes isolated from other countries during the outbreak period are in blue [34]. Sixty-four genomes belonging to subtypes other than subtype *emm*3.93 are represented in black (https://www.ncbi.nlm.nih.gov/datasets/genome/?taxon=301448).

Fig. 5 displays the phylogenetic tree based on core genome SNP analysis. Strains belonging to the same clade differed by < 10 SNPs [33]. The *emm*3.93 strains in this study differed by 40-100 SNPs from other *emm*3 subtypes, whereas differences with *emm*3.93 strains from other countries ranged 3-30 SNPs.

The ten *emm*3.93 strains were grouped into three clades. Seven strains formed the “Spanish Clade”, showing 0-6 SNPs differences among them; this clade included the four ΦspeC-spd1-positive-strains. A second clade grouped strains 20240174 and 20240285 -showing a chromosomal arrangement like MGAS315- and one strain from England (2023-2024). “Clade 3”, previously described by Davies et al. [33], clustered strain 20240005 and strains from England and The Netherlands (2023-2024).

## 4 Discussion

*S. pyogenes emm*3 is a major cause of iGAS in Spain and other template countries [[4], [5], [6]] and has been involved in large outbreaks worldwide [26,37,38]. However, *emm*3 was absent from the global iGAS outbreak following the COVID-19 pandemic (2022-2023) [[9], [10], [11]]. In 2023, SPIGAS detected a nationwide outbreak driven by an unexpected increase of the previously rare *emm*3.93 subtype. Concurrently, a sharp increase in the incidence of *emm*3 cases was observed in several European countries and the United States [33,39,40]. The upsurge of this uncommon subtype suggests either the emergence of a new hypervirulent *emm*3 clone, or the successful re-emergence of a previously described clone after the silent pandemic period. Here, we analyze the *emm*3.93 outbreak detected in Spain during 2023-2024.

The sample was geographically representative, with no significant differences by sex, and people at the extreme of age were the most vulnerable. We previously described that *emm*3 was significantly associated with respiratory transmission and scarlet fever [6]. Now, the association of *emm*3 with sepsis further supports the virulence of the *emm*3.93 subtype.

In Spain, *emm*3.1 was majority until 2016. First detected in 2017, *emm*3.93 gradually replaced *emm*3.1, until both subtypes were equitable by 2019 (SPIGAS, unpublished data). The scarce number of *emm*3 strains in 2020-2022 is consistent with the marked decline in iGAS during the COVID-19 pandemic period [7,8]. Subtype *emm*3.93 was responsible for the eight-month-long outbreak of this study, whereas other *emm*3 subtypes were sporadic (Fig. 1, Fig. 2). Several, largely hypothetical factors may explain the emergence of *emm*3.93, including increased host susceptibility due to limited immunity to a novel subtype [41], or the potential impact of amino acid changes in the M protein (Fig. 3) on immune evasion [26]. Further research is required to clarify the determinants underlying the success of this emergent *emm*3.93 subtype.

Three *emm*3.93 clones were involved in the outbreak according to molecular analysis (Fig. 4), while genomic analysis enabled characterization of determinants defining the currently circulating *emm*3.93 strains. The clone *emm*3.93/*spe*A-*spe*G-*ssa* was prevalent. The minority clone *emm*3.93/*spe*A-*spe*C-*spe*G-*ssa* showed a temporal distribution like the majority clone; although it carried the *spe*C and *spd*1 genes, which might confer an ecological advantage, it remained restricted to northeastern Spain. The clone *emm*3.93/*spe*G-*ssa* partially mirrored *emm*3.93/*spe*A-*spe*G-*ssa* and disappeared alongside the outbreak. The emergence of *spe*A-negative clones has previously been associated with the decline of *emm*3 GAS outbreaks [26,37], highlighting the role of SpeA in GAS virulence. However, we did not observe association between *speA*-positive clones and more severe clinical syndromes.

Antimicrobial susceptibility results are consistent with previous data from Spain which do not associate GAS *emm*3 with resistant clones [6]. Antimicrobial resistance was not determinant for the epidemic re-emergence of type *emm*3.

Two large chromosomal rearrangements have been described in GAS *emm*3: an inversion of >500 Kbp around the *ori*, and an inversion of approximately 200 Kbp around the *ter*. Taking the chromosomal arrangement of strain MGAS315 (*ori*-/*ter*-) as the consensus [12], these inversions were first described in strain SSI-1 (*ori*+/*ter*+) [13]. Recently, strains carrying only the *ori* inversion (*ori*+/*ter*-) or only the *ter* inversion (*ori*-/*ter*+) have been described [33]. In this study, we detected a minority arrangement like MGAS315 (*ori*-/*ter*-) and a majority arrangement carrying only the *ter* inversion (*ori*-/*ter*+). Our results support the hypothesis that the two inversions occur independently [26,31]. The *ter* inversion appears to be more frequent, although firm conclusions about the predominant chromosomal arrangements are limited by the sample size. Such inversions seem common in *emm*3 GAS and have also been described *in vitro* [26]. Their potential adaptive advantage remains unclear and warrants further investigation.

The six distinctive Φ315 prophages of *emm*3 are present and the less common ΦspeC-spd1 prophage has already been described in other *emm*3 epidemic lineages [26,38]. Prophage content is consistent with virulence gene profile. Φ315.1 carries *ssa* in *ter* + strains, and Φ315.2 carries *ssa* in *ter*-strains. Φ315.3, Φ315.4, Φ315.5 and Φ315.6 carry *spd*4, *spe*K and *sla*A, the *spe*A3 allele, and *sdn*, respectively [[12], [13], [14], [15], [16],^26^,^33^]. Limitations of molecular analysis explain discrepancy with WGS exotoxin results; *spe*K was not searched by PCR and *sme*Z was not detected because of its high allelic diversity.

The *nag*-*slo* operon is typical of hypervirulent *emm*3 strains circulating since earliest genomic studies [42]. The three polymorphisms defining promoter variant 3 enhance transcription of *nag* and *slo*, and the Asp330Gly amino acid substitution is related to higher NAD glycohydrolase activity. These changes increase the bacterial ability to kill host's cells [35,36].

Phylogenetic analysis clustered *emm*3.93 strains apart from other *emm*3 subtypes (Fig. 5). The “Spanish Clade” could be regarded the most representative of *emm*3.93 strains circulating during the outbreak in Spain; the *ter* inversion was universal, and it included strains carrying ΦspeC-spd1. Notably, a subset of strains clustered with *emm*3.93 isolates from other European countries [33], indicating that the spread of GAS *emm*3.93 resulted in an outbreak of international scope.

In conclusion, molecular and genomic analysis support that the *S. pyogenes* subtype *emm*3.93 outbreak detected in Spain during 2023-2024 was mainly driven by the clone *emm*3.93/*spe*A-*spe*G-speK-smeZ-*ssa*/ST315. The emergent *emm*3.93 subtype exhibits genomic features characteristic of contemporary hypervirulent *emm*3 strains. Continuous surveillance is essential to prevent and control the iGAS.

## Funding

This work was partially supported by the *Instituto de Salud Carlos III*, project MPY 289/25. MV was contracted via grant PEJ CAM 2021/TL/BMD-21100 from the *Programa Operativo Empleo Juvenil e Iniciativa Empleo Juvenil (YEI*).

## CRediT authorship contribution statement

**Villalón Pilar:** Conceptualization, Data curation, Formal analysis, Funding acquisition, Investigation, Methodology, Project administration, Resources, Supervision, Validation, Visualization, Writing – original draft, Writing – review & editing. **Medina-Pascual María José:** Data curation, Formal analysis, Investigation, Methodology, Validation, Writing – review & editing. **Garrido Noelia:** Data curation, Formal analysis, Methodology, Validation. **Valiente Mónica:** Data curation, Formal analysis, Methodology, Validation. **Monzón Sara:** Data curation, Formal analysis, Methodology, Supervision, Validation, Writing – review & editing. **Varona Sarai:** Data curation, Formal analysis, Methodology, Supervision, Validation. **Valdezate Sylvia:** Data curation, Formal analysis, Funding acquisition, Investigation, Methodology, Validation, Writing – review & editing.

## Declaration of competing interest

The authors declare that they have no known competing financial interests or personal relationships that could have appeared to influence the work reported in this paper.

## Data Availability

Raw sequence data and this Whole Genome Shotgun project have been deposited at DDBJ/ENA/GenBank under the bioproject PRJNA1358835, accessions SAMN53128930 (https://www.ncbi.nlm.nih.gov/biosample/53128930), SAMN53128931 (https://www.ncbi.nlm.nih.gov/biosample/53128931), SAMN53128932 (https://www.ncbi.nlm.nih.gov/biosample/53128932), SAMN53128933, (https://www.ncbi.nlm.nih.gov/biosample/53128933), SAMN53128934 (https://www.ncbi.nlm.nih.gov/biosample/53128934), SAMN53128935, (https://www.ncbi.nlm.nih.gov/biosample/53128935), SAMN53128936 (https://www.ncbi.nlm.nih.gov/biosample/53128936), SAMN53128937 (https://www.ncbi.nlm.nih.gov/biosample/53128937), SAMN53128938 (https://www.ncbi.nlm.nih.gov/biosample/53128938) and SAMN53128939 (https://www.ncbi.nlm.nih.gov/biosample/53128939).
