# Supplementary material for: Sudden re-emergence of Streptococcus pyogenes subtype emm3.93 in Spain, 2023-2024
Source: New Microbes New Infect. 2026 Jun 13;72:101794. doi: 10.1016/j.nmni.2026.101794 (PMC13292671; doi:10.1016/j.nmni.2026.101794)
Supplement: Multimedia component 1 [file mmc1.docx]

Supplementary Table 1. Temporal distribution of *S. pyogenes* type *emm*3 clinical syndromes in Spain, 2020-2024

| Clinical syndrome | 2020-2022 y-period  No. cases | 2023-2024 y-period  No. cases | Total cases |
| --- | --- | --- | --- |
| Sepsis | 2 | 62 | 64 |
| Pneumonia | 0 | 25 | 25 |
| Otitis | 0 | 19 | 19 |
| Abscess | 0 | 16 | 16 |
| OAI | 0 | 10 | 10 |
| Pharyngitis | 0 | 7 | 7 |
| STSS | 1 | 6 | 7 |
| Cellulitis | 0 | 6 | 6 |
| Puerperal fever | 0 | 5 | 5 |
| NF | 0 | 3 | 3 |
| Meningitis | 0 | 3 | 3 |
| Mastoiditis | 0 | 2 | 2 |
| Scarlet fever | 0 | 2 | 2 |
| Peritonitis | 0 | 1 | 1 |
| SWI | 0 | 1 | 1 |
| Vaginitis | 0 | 1 | 1 |
| Ulcer | 1 | 0 | 1 |
| Total | 4 | 169 | 173 |

Abbreviations: OAI, osteoarticular infection; STSS, streptococcal toxic shock syndrome; NF, necrotizing fasciitis; SWI, surgical wound infection.
